# Phytochemical Study of *Eryngium cymosum* F. Delaroche and the Inhibitory Capacity of Its Main Compounds on Two Glucose-Producing Pathway Enzymes

**DOI:** 10.3390/plants11070992

**Published:** 2022-04-05

**Authors:** Adriana Romo-Pérez, Sonia Marlen Escandón-Rivera, Luis D. Miranda, Adolfo Andrade-Cetto

**Affiliations:** 1 Laboratorio de Etnofarmacología, Departamento de Biología Celular, Facultad de Ciencias, Universidad Nacional Autónoma de México, Av. Universidad 3000, Circuito Exterior S/N, Coyoacán, C.U., Mexico City 04510, Mexico; adriana.romo@iquimica.unam.mx (A.R.-P.); soniaer@ciencias.unam.mx (S.M.E.-R.); 2 Laboratorio de Síntesis Orgánica, Instituto de Química, Universidad Nacional Autónoma de México, Av. Universidad 3000, Circuito Exterior S/N, Mexico City 04510, Mexico; lmiranda@unam.mx

**Keywords:** *Eryngium cymosum*, acylated flavonol glucoside, phenolic compounds, type 2 diabetes, glucogenesis inhibition, medicinal plants

## Abstract

One undescribed acylated flavonol glucoside and five known compounds were isolated from the aerial parts of *Eryngium cymosum* F. Delaroche, a plant that is used in traditional Mexican medicine to treat type 2 diabetes. The chemical structures of the isolated compounds were elucidated using a variety of spectroscopic techniques, including 1D and 2D nuclear magnetic resonance (NMR) and mass spectrometry (MS). Chlorogenic acid (**1**), rosmarinic acid (**2**), caffeic acid (**3**), protocatechuic acid (**4**), kaempferol-3-*O*-(2,6-*di*-*O*-*trans*-*ρ*-coumaryl)-*β*-d-glucopyranoside (**5**), and the new acylated flavonol glucoside quercetin-3-*O*-(2,6-*di*-*O*-*trans*-*ρ*-coumaryl)-*β*-d-glucopyranoside (**6**) were isolated. This is the first report on the natural occurrence of quercetin-3-*O*-(2,6-*di*-*O*-*trans*-*ρ*-coumaryl)-*β*-D-glucopyranoside (**6**). In addition, according to the HPLC profile obtained for the water extract (WE), chlorogenic acid (**1**) and rosmarinic acid (**2**) were identified as the main compounds, while kaempferol-3-*O*-(2,6-*di*-*O*-*trans*-*ρ*-coumaryl)-*β*-d-glucopyranoside (**5**) were the main compound in the butanolic extract. We demonstrate the important role of compound **5** over the inhibition of G6Pase and FBPase. The isolated compounds may play an important role in the hypoglycemic effect of the extract and may act in a synergic way, but more experiments are needed to corroborate these findings.

## 1. Introduction

The *Eryngium* genus comprises approximately 250 species that are widely distributed worldwide (mainly in Eurasia, North Africa, and South America) and is the largest and most complex genus in the Apiaceae family [1]. Several *Eryngium* species have been used as antitussive, diuretic, appetizer stimulant, anti-inflammatory, aphrodisiac, anti-diarrheic, and hypoglycemic agents and as a remedy for scorpion stings [2]. *Eryngium cymosum* F. Delaroche, commonly called “piñuela”, is a perennial herb that is located in some states in Mexico, such as Hidalgo, Guerrero, Michoacán, and Mexico State [3].

Type 2 diabetes (T2D) is one of the fastest growing global health emergencies of the 21st century, as defined by the International Diabetes Federation (IDF), and is a chronic condition that occurs when blood glucose levels are increased; in this condition, the body cannot produce any or enough insulin or cannot effectively use the insulin it produces [4]. The IDF ranked Mexico as having the 7th largest numbers of adults with diabetes in 2021. These individuals must focus on achieving the optimal glycemic control to avoid diabetic complications; in addition, it was observed that during the first wave of coronavirus disease 2019 (COVID-19), individuals with diabetes had a 3.6-fold higher likelihood of being hospitalized due to COVID-19 than those without diabetes [4], so glycemic control in T2D is a concern throughout the world.

In Mexico, for historical and cultural reasons, medicinal plants are still used for different diseases [5], including T2D. We documented that in the community of Tlanchinol, Hidalgo, individuals with T2D control their glucose levels with decoctions of the aerial parts of medicinal plants [6]. In a previous work, our group demonstrated that the aqueous extract of aerial *Eryngium cymosum* plant parts exert a hypoglycemic effect in hyperglycemic streptozotocin and nicotinamide (STZ-NA) rats. As the main mechanism of action, the extract inhibited endogenous glucose production, specifically gluconeogenesis, which is the impaired pathway that causes fasting and postprandial hyperglycemia in T2D patients. The suppressive ability of the extract was potent and effective against the key gluconeogenic enzymes glucose-6-phosphatase (G6Pase) and fructose-1,6-bisphosphatase (FBPase). The extract did not present an inhibition effect on intestinal α-glucosidases or on acute insulin rates, but it increased the insulin sensitivity index in OGTTs; furthermore, three compounds were identified [6].

In the current study, we aimed to isolate the main compounds from the water extract (WE), methanolic extract (ME), and organic extract (Dicloromethane:metanol extraction, OE) of *Eryngium cymosum* by chromatographic column. Six compounds (**1**–**6**), including one new compound, quercetin-3-*O*-(2,6-*di*-*O*-*trans*-*ρ*-coumaryl)-*β*-d-glucopyranoside (**6**), were isolated from the *Eryngium* extracts. Herein, we describe the isolation and structural determination of Compounds **1**–**6.** Moreover, since we previously observed the ability of the extract to inhibit endogenous glucose production, specifically gluconeogenesis, compounds; the more abundant in the OE and one present in both extracts were tested in vitro on two of the rate-limiting gluconeogenic enzymes (glucose-6-phosphatase (G6Pase) and fructose-1,6-bisphosphatase (FBPase)) to see if they are directly involved in this mechanism of action. This research improves our understanding of the chemical composition of the plant and a possible bioactive compound.

## 2. Results

### 2.1. Extraction and Isolation

The WE, ME, and OE from the aerial parts of *Eryngium cymosum* were phytochemically investigated for the first time in this study. The percent yields of aqueous, methanolic, and organic extraction were 15.5%, 12.2%, and 3.8% (*w*/*w*), respectively.

**Figure 1:**
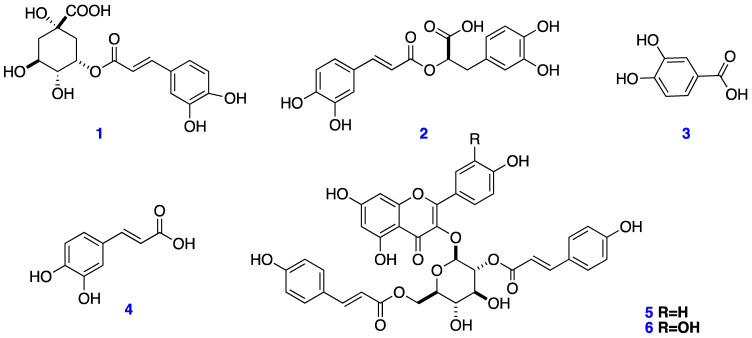
Compounds isolated from *Eryngium cymosum.* Chlorogenic acid (**1**); rosmarinic acid (**2**); protocatechuic acid (**3**); caffeic acid (**4**); kaempferol-3-*O*-(2,6-*di*-*O*-*trans*-*ρ*-coumaryl)-*β*-d-glucopyranoside (**5**); and quercetin-3-*O*-(2,6-*di*-*O*-*trans*-*ρ*-coumaryl)-*β*-d-glucopyranoside (**6**).

The WE from *Eryngium cymosum* underwent chromatographic fractionation, resulting in the isolation of two known compounds, chlorogenic acid (**1**) [7] and rosmarinic acid (**2**) [8], which were the major compounds present in the extract. On the other hand, when BuS (butanolic subfraction, see Section 4.3) underwent chromatographic isolation, protocatechuic (**3**) and caffeic (**4**) acids [9], and the flavonol kaempferol-3-*O*-(2,6-*di*-*O*-*trans-ρ*-coumaryl)-*β*-d-glucopyranoside (**5**), were isolated [10]. The previous metabolites (**1**–**5**) were identified by comparing their spectroscopy data with those previously reported in the literature [7,8,9,10] and the chemical structures are shown in Figure 1. Finally, in the OE extract, the acylated flavonol glucoside **5** and the new Compound **6** were isolated, and their spectroscopic data are presented in Table 1.

### 2.2. Structural Elucidation of Compound **6**

Compound **6** was obtained as a yellow amorphous powder, which revealed a molecular ion peak at *m*/*z* 738 [M − H_2_O]^−^, which would correspond to the expected mass of compound *m*/*z* 756 minus one molecule of water (Figure S9). The infrared (IR) spectrum of **6** showed the presence of OH (3254 cm^−1^), C-H (2924 and 2854 cm^−1^), C=O (1625 cm^−1^), and the phenyl rings (1571 and 1485 cm^−1^) (Figure S10). The ^1^H-NMR (Figure S4) of **6** exhibited an aromatic ABX type signals at *δ*_H_ 7.55 (dd, *J* = 8.6, 2.1 Hz), 6.85 (d, *J* = 9 Hz), and 7.56 (d, *J* = 2.1 Hz) assignable to H-6′, H-5′, and H-2′, respectively, which, according to the HSQC experiment (Figure S6), correspond to the carbons that resonate C2′ (117.1), C5′, (116.0) and C6′ (123.5). Additionally, the aromatic region exhibited a typical *meta*-coupled pattern for H-6 (*δ*_H_ 6.27, *J* = 2.1 Hz) and H-8 (*δ*_H_ 6.09, *J* = 1.8 Hz) protons that are in the A ring of flavonol. Furthermore, in the ^1^H-NMR spectrum (Figure S4), characteristic signals of the presence of a sugar are observed, when observing the anomeric proton that resonates at *δ*_H_ 5.63 (d, *J* = 8.0 Hz, H-1″), which corresponds, according to the HSQC experiment (Figure S6), to carbon *δ*_C_ 100.4 and five signals in a range of *δ*_H_ 3.46 and 5.13 ppm. The coupling constant (*J* = 8.0 Hz) of the anomeric proton H-1″ was indicative of the *β*-form glucose.

**Figure 2:**
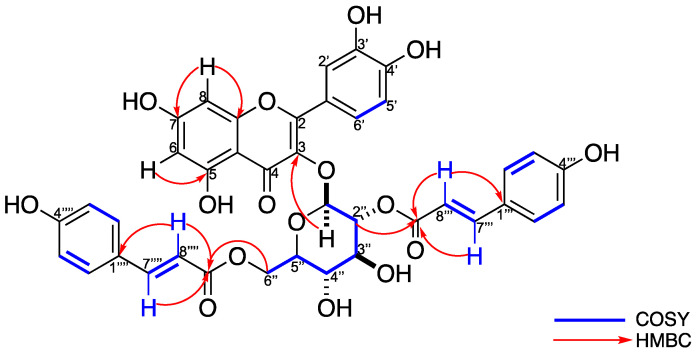
COSY and HMBC correlations for **6**.

Thereby, the sugar of compound **6** was confirmed as *β*-D-glucopyranose. Furthermore, in ^1^H- NMR (Figure S4), four double signals are observed at *δ*_H_ 7.73 (*J* = 15.9 Hz), 6.44 (*J* = 15.9 Hz), 7.42 (*J* = 15.9 Hz), and 6.10 *(J* = 15.9 Hz) with a characteristic coupling constant of *trans*-olefinic protons and the COSY correlation are of the protons that resonate at *δ*_H_ 7.73–6.44 and 7.42–6.10 (Figure 2 and Figure S5). Furthermore, in the ^1^H-NMR spectrum, two AA′BB′ systems are observed, corresponding to the doublets observed at *δ*_H_ 7.50 (*J* = 8.7 Hz, H-2‴, H-6‴) and 6.83 (*J* = 8.6 Hz, H-3‴, H-5‴) for the first system, and 7.33 (*J* = 8.6 Hz, H-2⁗, H-6⁗) with 6.83 (*J* = 8.6 Hz, H-3⁗, H-5⁗) for the second system (Figure S4). These protons correspond to the strong carbon signals seen at *δ*_C_ 131.2 and 116.8 for the first system, and *δ*_C_ 131.3 and 116.8 for the second AA′BB′ system (Figure S6). Furthermore, in the ^13^C-NMR (Figure S8), two carbonyls are observed that resonate at δ_C_ 168.6 and 168.8, which, according to the HMBC experiment (Figure 2 and Figure S7), correlate with the protons that resonate at *δ*_H_ 7.73 and 6.44 for the carbonyl that resonates at *δ*_C_ 168.6, and *δ*_H_ 7.42 and 6.10 for the carbonyl that resonates at *δ*_C_ 168.8. This indicates the presence of two cinnamic moieties. According to the HMBC experiment (Figure 2 and Figure S7), a cinnamic molecule is in position 2 of the sugar, since a correlation of the carbonyl that resonates at *δ*c 168.6 with the H-2″ (5.13, dd, *J* = 9.5, 8.0 Hz) of the sugar. The other cinnamic molecule present in compound **6** is bound, according to the HMBC experiment, to the C-6″ (*δ*_C_ 64.2) of the sugar, observing a correlation of the carbonyl group that resonates at *δ*_C_ 168.7 with the H-6a″ (4.38, dd, *J* = 11.7, 2.2 Hz) and H-6b″ (4.22, dd, *J* = 11.8, 6.8 Hz) protons (Figure 2 and Figure S7). The ^1^H- and ^13^C-NMR data (Table 1) were in complete agreement with the proposed structure and compound **6** was established as quercetin-3-*O*-(2,6-*di*-*O*-*trans*-*ρ*-coumaryl)-*β*-d-glucopyranoside (Figure 2).

### 2.3. HPLC Profiles

**Figure 3:**
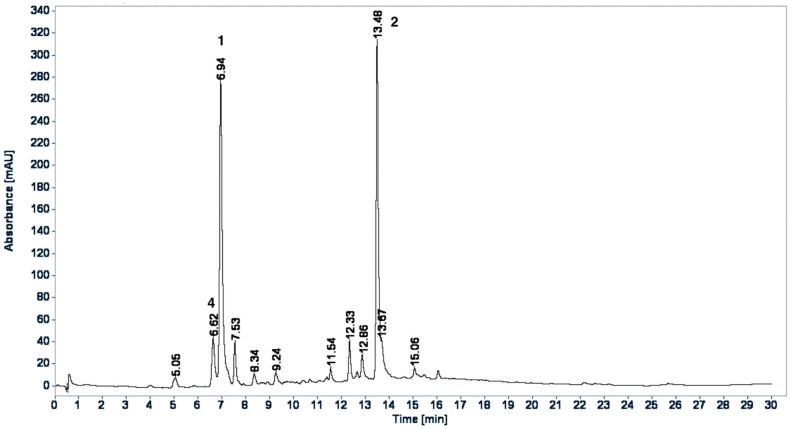
HPLC-DAD profile of water extract of *E. cymosum*; acquisition of λ 320 nm. Phenomenex^®^ Kinetex C_18_ (Phenomenex Inc., Torrance, CA, USA) 100 Å (50 × 2.1 mm id., 5 μm) reversed-phase column; the column temperature was kept at 35 °C; the flow rate was 0.35 mL/min; elution was carried out with water containing 0.1% (*v*/*v*) formic acid and acetonitrile (MeCN), as described in the experimental section.

**Figure 4:**
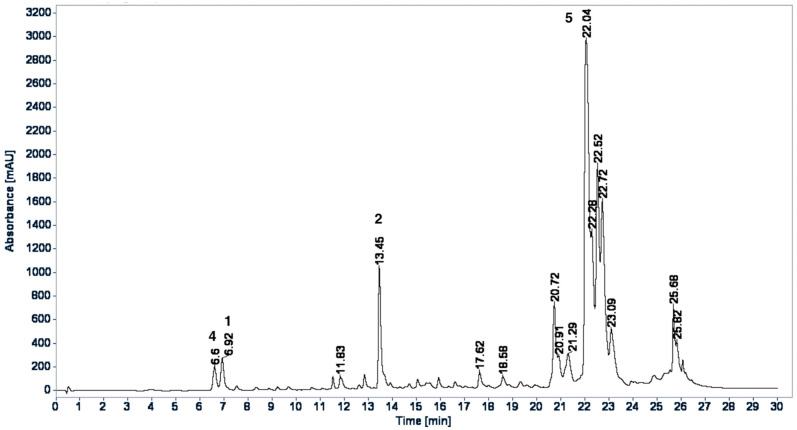
HPLC-DAD profile butanol extract of *E. cymosum*; acquisition of λ 320 nm. Phenomenex^®^ Kinetex C_18_ (Phenomenex Inc., Torrance, CA, USA) 100 Å (50 × 2.1 mm id., 5 μm) reversed-phase column; the column temperature was kept at 35 °C; the flow rate was 0.35 mL/min; elution was carried out with water containing 0.1% (*v*/*v*) formic acid and acetonitrile (MeCN), as described in the experimental section.

HPLC profiles for WE and BuS were developed to qualitatively identify the proportion of isolated compounds. In the chromatographic HPLC profiles of the WE (Figure 3) and BuS (Figure 4) extracts, most components showed maximum absorption at 320 nm. The compounds chlorogenic acid (**1**), rosmarinic acid (**2**), and caffeic acid (**4**) were identified in the extracts by coelution with the standards in HPLC and by comparing retention time and UV spectrum data of the peaks that were identified in the chromatographic profiles; in comparison, Compound **5** was identified by being coeluted with the extracts and by comparing UV spectrum data and retention time of the peak in the chromatographic profiles. Because Compounds **3** and **6** were not abundant in the extracts, there was a lack of standards and the samples rapidly decomposed; these compounds were not identified in the extracts. The two chromatograms were qualitatively different; however, the main components of the WE [T_R_: 6.62 (**4**), 6.94 (**1**), and 13.48 (**2**)] were also observed in BuS [T_R_: 6.60 (**4**), 6.92 (**1**), and 13.45 (**2**)], but in different proportions, and **5** was the major compound in this extract (Figure 4).

### 2.4. In Vitro Inhibitory Assays

The results of the inhibition assays of glucose-6-phosphatase (G6Pase) and fructose-1,6-bisphosphatase (FBPase) are shown in Table 2. The isolated caffeic acid (**4**) showed a modest inhibition over G6Pase and no inhibition over FBpase, while kaempferol-3-*O*-(2,6-*di*-*O*-*trans*-*ρ*-coumaryl)-β-d-glucopyranoside (**5**) showed an important inhibition over the two enzymes, suggesting that this compound could be involved in the hypoglycemic effect of the plant.

## 3. Discussion

In this study, we described the phytochemical composition of the aerial parts of *Eryngium cymosum*, a plant commonly used in Tlanchinol, Hidalgo, Province of Mexico, for the management of T2D mellitus.

Secondary metabolites, such as terpenoids, triterpenoid glycosides, coumarins, flavonoids, acetylenes, and essential oils have been reported in the genus *Eryngium*. Compounds **1**, **2,** and **4** have been identified in species such as *E. alpinum*, *E. planum*, and *E. bourgatii* [11,12,13]. A study by Shing et al. [11] reported the presence of **2** in at least 15 species. Compound **3** has been identified in methanolic extracts from *E. foetidum* and *E. serbicum* [14,15]. Flavonol **5** was previously isolated from *E. yuccifolium* and *E. caeruleum* [10,16].

Phenolic acids are naturally occurring compounds, are found in the plant kingdom, have unique structural similarities, and contain carboxylic groups similar to those in the phenolic compounds isolated in this work [17].

Several reports have shown the wide biological activities of rosmarinic acid, including astringent, antioxidative, anti-inflammatory, antimutagen, antibacterial, antiviral, cytotoxic, neuroprotective, and hypoglycemic [18,19,20]. In a study conducted in a model of STZ-induced diabetic rats with a high carbohydrate diet, it was found that after 4 days of ad libitum consumption of rosmarinic acid (**2**), the blood glucose levels were decreased by inhibiting the sodium-glucose transporters SGLT1 [21]. In their work, Jayanthy and Subramanian demonstrated the potential hypoglycemic activity of rosmarinic acid (**2**) in high-fat diet model-STZ-induced rats after consumption for 30 days, and they observed a decrease in blood glucose levels and an increase in plasma insulin. In addition, they observed a decrease in activity for the enzymes involved in carbohydrate metabolism and an increase in activity for the enzymes involved in glycogen metabolism. The values were similar to those observed in rats that were treated with metformin, so the researchers suggested that rosmarinic acid (**2**) could be considered for use in the treatment of type 2 diabetes [20].

Chlorogenic acid (**1**), also known as 5-*O*-caffeoylquinic acid, is a phenolic compound that is commonly found in plants, and it is produced by the esterification of a C6-C3 *trans*-hydroxycinnamic acid (caffeic acid) with quinic acid. This is the most abundant isomer and is found in many foods, such as coffee and tea [22]. Chlorogenic acid (**1**) has been studied extensively, and there is evidence of its various biological activities, including antibacterial, antioxidant, anti-inflammatory, antihypertensive, anti-obesity, and hypoglycemic [23]. Several studies have found that chlorogenic acid (**1**) lowers blood glucose levels by decreasing the release of liver glucose and by decreasing postprandial glucose by inhibiting the enzymes α-glucosidases and α-amylases. In addition, it has been demonstrated that **1** stimulates insulin release in the rat islets and also that it is a novel insulin sensitizer [23,24].

Protocatechuic acid (**3**), also known as 3,4-dihydroxybenzoic acid, is a phenolic compound and is found in foods that are consumed by humans, such as plums, grapes, nuts, and almonds, and in products with plant origins, such as olive oil and white wine. Protocatechuic acid (**3**) has been reported for its biological activities, such as antioxidant, antibacterial, anticancer, antiulcer, antiviral, and antidiabetic activities, among others [17]. The hypoglycemic effect of protocatechuic acid (**3**) was demonstrated in STZ-induced diabetic rats after oral administration for 45 days, where there was a decrease in plasma glucose and an increase in plasma insulin at a dose of 100 mg/kg bw [25].

Caffeic acid (**4**) is a hydroxycinnamic acid compound that is mostly distributed in the plant kingdom. This compound has been studied extensively and mainly for its antioxidant properties; however, there are reports that it presents hypoglycemic activity. The group of Oboh et al. reported the inhibitory effect on α-amylase and α-glucosidase, and the results showed a dose-dependent inhibition, which was more selective to α-amylase (IC_50_ = 3.68 μg/mL) than α-glucosidase enzyme (IC_50_ = 4.98 μg/mL) [26]. In addition, in a study conducted in a *db*/*db* mouse model treated with caffeic acid (**4**) for 5 weeks, it was found that plasma glucose levels decreased, and blood insulin levels increased. Additionally, they observed an increase in the activity of the enzyme glucokinase and a decrease in the activity of glucose-6-phosphatase and PEPCK. In the histological study of the pancreas of mice treated with caffeic acid (**4**), the islets were preserved. Otherwise, in the pancreas of the control group, the islets were very affected. Therefore, the antidiabetic activity combined with the known antioxidant activity of caffeic acid could help prevent or slow the development of diabetes [27].

Flavonoids are polyphenolic compounds that are widely distributed in the plant kingdom and can be found in their free form (aglycone), glycosides and methylated derivatives. Flavonols are flavonoids that have a ketone group and a hydroxyl group at position 3 of ring C, which can be glycosylated and are widely distributed in fruits and vegetables. The best known flavonols are kaempferol and quercetin and their derivatives [28]. Its antioxidant, anti-inflammatory, antiviral, anticancer, and hypoglycemic properties are recognized [29]. In a study conducted in 2016, the inhibitory activity of aldose reductases (ALR-1 and ALR-2) of two glycoside flavonols esterified with coumaric acid was determined. The results showed that the doubly esterified flavonol showed a higher IC_50_ than that of the two aldose reductases (ALR1 = 1.31 ± 0.67 μM and ALR2 = 0.93 ± 0.67 μM) compared to that of flavonol, which has only one coumaric acid ester in its structure (ALR1 = 12.22 ± 4.32 and ALR2 = 2.54 ± 0.98). In addition, compared to the positive controls, including valproic acid (ALR-1, 57.4 ± 0.89 μM) and sorbinil (ALR-2, 3.14 ± 0.02 μM), these compounds were more active on the enzymes ALR-1 and ALR-2 [10].

Some species from the *Eryngium* genus have been studied for their hypoglycemic activity, including the following: the oral administration of ethanolic extract of *Eryngium carlinae* was studied in streptozotocin-induced diabetic rats for over 40 days of administration at doses of 30 mg/kg. The results showed that the extract has no hypoglycemic activity. However, the biochemical parameters demonstrated their hypolipidemic effect. Therefore, the authors suggest that *E. carlinae* can be used as an adjuvant in the treatment of diabetes by delaying the complications that occur in this disease [30]. On the other hand, *Eryngium creticum*, a species that is used in traditional Jordanian medicine as an antidote for scorpion poison and its hypoglycemic effect, has been reported in in vitro and in vivo assays in Jordan. The Jaghabir group demonstrated that the aqueous extract of *E. creticum* decreases blood glucose levels by 31% at 4 h after treatment and up to 62% after 24 h of treatment in STZ-induced diabetic rats [31]. Additionally, Kazabri et al. evaluated the in vitro insulinotropic activity of the aqueous extract of *E. creticum* in MIN6 cells, and they observed that there was glucose-stimulated insulin secretion (GSIS) at a dose of 0.01 mg/mL aqueous extract [32]. In addition, they demonstrated that *Eringium creticum* does not have an inhibitory effect on α-amylase and α-amyloglucosidase enzymes in vitro, but it does show acute hyperglycemic activity after 45 min of oral administration of the aqueous extract (125–500 mg/kg) in normal rats after an oral starch tolerance test [33]. The enzymatic inhibitory activity (α-glucosidase and α-amylase) of the ethanolic, acetone, and aqueous extracts of *Eryngium bornmuelleri* was reported. According to the results, the organic extracts had an IC_50_ higher than that of the aqueous extract and are attributed to the presence of phenolic compounds that are present in the organic extracts [34]. *E. foetidum*, an Indian plant used in traditional medicine to treat pain, fever, constipation, asthma, arthritis, and diarrhea, was evaluated by Malik et al. for its antihyperglycemic activity. For this, they tested the ethanolic, methanolic, and aqueous extracts in an enzyme inhibition assay with α-amylase in vitro. The results showed that the ethanolic extract exhibited greater inhibitory activity (46%) than that of the methanolic (52%) and aqueous (30%) extracts [35].

As we mentioned, in the HPLC profiles, compounds protocatechuic acid (**3**) and quercetin-3-*O*-(2,6-*di*-*O*-*trans*-*ρ*-coumaryl)-*β*-d-glucopyranoside (**6**) were not abundant in the extracts, so it is less likely that they are involved in the hypoglycemic effect related to the inhibition of the hepatic glucose output via enzymatic inhibition of the gluconeogenesis pathway, while this action is widely demonstrated for chlorogenic acid (**1**) [24], for rosmarinic acid (**2**) [20], and, in a lesser way, for caffeic acid (**4**) [27]. We demonstrated that the isolated caffeic acid (**4**) had a modest inhibition over the G6Pase and no inhibition over FBPase. These results are in agreement with [27]. Furthermore, we found that kaempferol-3-*O*-(2,6-*di*-*O*-*trans*-*ρ*-coumaryl)-*β*-d-glucopyranoside (**5**) showed an important inhibition over the two enzymes, suggesting that this compound could be involved in the hypoglycemic effect of the plant.

The presence of these reported phenolic acids and flavonols in *Eryngium cymosum*, especially compound (**5**), could explain its hypoglycemic activity, while the use of other *Eryngium* species as hypoglycemic agents reinforce the use of some members of the genus to treat type 2 diabetes.

## 4. Materials and Methods

### 4.1. General Experimental Procedures

Compounds were monitored by thin-layer chromatography (TLC) on 0.25-mm silica gel plates and were visualized under UV light or through ceric ammonium sulfate and vanillin staining. Column chromatography separations were performed using Sephadex LH-20 (Sigma-Aldrich Chemical) and silica gel 60 (particle size 230–400 mesh, Sigma-Aldrich). The solvent mixtures employed in TLC analysis and column chromatography purification are reported as volume by volume. 1D and 2D spectra were run on Bruker Ascend 700 MHz and 500 MHz Bruker Avance III spectrometers with tetramethylsilane as an internal standard and acetone-d_6_ (CD_3_COCD_3_), methanol-d_4_ (CD_3_OD), and water-d_2_ (D_2_O) as the solvents. Low-resolution mass spectra (LRMS) were recorded on JEOL SX102A (Jeol, Ltd. Tokyo, JP) and Bruker Daltonics Esquire 6000 (Bruker Daltonics, Billerica, MA, USA) mass spectrometers. Analytical HPLC analyses were performed on an Agilent 1260 Infinity (Agilent Technologies Inc. Santa Clara, CA, USA) system equipped with a G1311B quaternary pump (Agilent Technologies Inc. Santa Clara, CA, USA), a G1367E autosampler (Agilent Technologies Inc. Santa Clara, CA, USA), and a G1315C DAD (Agilent Technologies Inc. Santa Clara, CA, USA). Data acquisition and processing and management of the chromatographic information were performed by OpenLab CDS ChemStation Edition (version A.01.06.11, Agilent Technologies, Santa Clara, CA, USA) (2001–2013) software. All solvents were purchased from JT Baker (Avantor Performance Materials, S.A. de C.V., Xalostoc, MX) and were HPLC grade. Standards of caffeic (C0625), chlorogenic (C3878), and rosmarinic (R4033) acids were purchased from Sigma-Aldrich (Steinheim, Germany).

### 4.2. Plant Material

The aerial parts of *Eryngium cymosum* Delaroche were collected in Huejutla de Reyes, state of Hidalgo, Mexico in 2017. A voucher specimen (ETNOF248) was deposited at the FC herbarium. New and fresh plant material were collected as needed.

### 4.3. Extraction and Isolation

The water extract (WE) was prepared by boiling 20 g of the dry plant material with 500 mL water, followed by filtration and lyophilization to yield 3.1 g of WE. The extract was stored at 4 °C for further analysis.

For the identification of the main compounds in the aerial parts, the ME was prepared using 110 g of powdered *E. cymosum* aerial parts through Soxhlet extraction. The pulverized plant was defatted with n-hexane (nH) (48 h) and then extracted (48 h) with methanol (MeOH). The resulting extract was evaporated under reduced pressure until dry, producing 13.4 g of the ME, which was refrigerated for further studies. A portion of ME (5.5 g) was resuspended in CH_3_OH:H_2_O (80:20, 250 mL) and partitioned with CCl_4_ (250 mL). The hydroalcoholic extract was evaporated under reduced pressure until it was dry. The extract was solubilized with water and extracted with butanol (200 mL) to obtain the butanolic subfraction (BuS, 1.2 g).

The OE was prepared from 450 g of powdered plant material by consecutive extraction with a mixture of dichloromethane:methanol (1:1, 48 h, two times) that was previously defatted with hexane (48 h). The combined OE extract was concentrated in a rotary vacuum evaporator at 40 °C; the resulting concentrate was evaporated to dryness under reduced pressure, yielding 17.0 g of OE.

The water, methanolic extract, and organic extract were used in the phytochemical identification of the main compounds of *Eryngium cymosum.*

The WE (3.0 g) was solubilized in a mixture of H_2_O:CH_3_OH (50:50), separated on Sephadex LH-20 (GE Healthcare, Chicago, IL, USA) CC (60 g, 16 cm length × 3.5 cm diameter), and eluted with H_2_O:CH_3_OH (50:50 and 0:100) to give 7 fractions (EW1–EW7). Fractions EW5 (95 mg) were subjected to LH-20 CC using H_2_O:CH_3_OH (50:50, 30:70 and 0:100) as the eluent to obtain the following 13 eluates: 1–4 (eluates were discarded), 5–6 (EW5.1), 7–10 (EW5.2), and 11–13 (eluates were discarded). In subfraction EW5.1, 4.8 mg of **1** was obtained, and in EW5.2 (35 mg), a mixture of **1** and **2** was observed. Therefore, this subfraction was combined with EW6 (39 mg), subjected to Sephadex LH-20 CC, and eluted with H_2_O:CH_3_OH (50:50 to 0:100) to obtain 26 eluates, which were pooled into 4 subfractions (EW6.1–EW6.4). Eluates 1–15 were discarded, and the others were collected as follows: EW6.1 (16–19), EW6.2 (20–21), EW6.3 (22), EW6.4 (23–24). Compound **1** was isolated in the subfractions EW6.1 (15.7 mg) and EW6.2 (8.9 mg), and in the subfractions EW6.3 (5.3 mg) and EW6.4 (21.6 mg), Compound **2** was isolated. Finally, the EW7 subfraction (45 mg) was separated by Sephadex LH-20 CC using H_2_O:CH_3_OH (50:50 to 0:100) as the mobile phase, and 18 eluates were obtained. According to the thin layer chromatography screening, eluates 1–8 were discarded, and the other eluates were pooled by chromatographic similarity into the following 3 subfractions: EW7.1 (9–11), EW7.2 (12–14), and EW7.3 (15–18). Compound **2** was isolated from the EW7-1 subfraction (17.2 mg).

A portion of the ME (5.12 g) was chromatographed on 60 g Sephadex LH-20 CC (16 cm length × 3.5 cm diameter) and eluted with MeOH. A total of 57 fractions (30 mL each) were collected and monitored by TLC. These fractions were pooled into the following 7 collective fractions (ME1–ME7) based on similar TLC profiles: eluates 1–9 (ME1), eluates 10–21 (ME2), eluates 22–42 (ME3), eluates 43–48 (ME4), eluate 49 (ME5), eluate 50 (ME6), and eluates 51–57 (ME7). ME5 (116 mg) was subjected to silica gel CC (2.5 g) via a gradient elution with EtOAc:CH_3_OH (from 100:0 to 0:100) to afford 27 fractions that were pooled into 3 collective fractions (ME5.1–ME5.3). Preparative TLC (CHCl_3_:CH_3_OH, 85:15, 50 mL) of fraction ME5.3 (45 mg) resulted in the isolation of flavonol glycoside **5** (7 mg) and a mixture of Compounds **1** and **2** (20 mg). This mixture was purified by preparative TLC (EtOAc:MeOH: H_2_O, 80:15:5, 50 mL) to obtain Compounds **1** (4.1 mg) and **2** (6.9 mg).

On the other hand, the butanolic subfraction (BuS, 1.0 g) underwent silica gel CC (20 g) using hexanes:EtOAc (from 50:50 to 0:100) and EtOAc:CH_3_OH (from 95:05 to 0:100) to produce 30 fractions (20 mL each). These fractions were pooled into 6 fractions (BuS1–BuS6) on the basis of similar TLC profiles. BuS3 (289 mg) was separated by silica gel CC (1.5 g) using hexanes:acetone (from 9:1 to 0:1) to obtain 34 subfractions, which were pooled into 5 collective fractions (BuS3A to BuS3E). BuS3C (289 mg) was separated by silica gel CC (5.0 g) using hexanes:acetone (from 9:1 to 0:1) to obtain 11 eluates. These eluates were pooled into 3 subfractions (BuS3C-1 to BuS3C-3). Subfraction BuS3C-2 was separated by preparative TLC (10 × 20 cm) and eluted with CH_2_Cl_2_:CH_3_OH (9:1, 40 mL) to isolate Compounds **3** and **4** as a mixture (7 mg). Additionally, BuS3D (86 mg) was separated by preparative TLC (20 × 20 cm) and eluted with CH_2_Cl_2_:CH_3_OH OH (9:1, 50 mL) to obtain **5** (12 mg).

The OE extract (6.0 g) was subjected to fractionation using silica gel (65 g) as a stationary phase and was eluted with hexanes:EtOAc (50:50, 30:70) and EtOAc:CH_3_OH (95:05, 0:100) systems to obtain 22 fractions (50 mL each). These fractions were pooled into 7 fractions (OE1–OE7) based on similar TLC profiles. OE2 (217 mg) was subjected to silica gel column chromatography (3.5 g) and eluted with a gradient of hexanes:EtOAC (from 8:2 to 0:1) to obtain 5 subfractions (EO2.1–EO2.5). Subfraction EO2.2 (157 mg) was subjected to column chromatography with a gradient elution of CH_2_Cl_2_:CH_3_OH (from 100:0 to 1:1) and silica gel as a stationary phase (3.0 g), and we obtained 24 eluates (8 mL each) that were pooled into 5 subfractions (EO2.2.1–EO2.2.5) on the basis of similar TLC profiles. Subfraction EO 2.2.3 (27 mg) was separated by preparative TLC (CH_2_Cl_2_:CH_3_OH, 9:1, 50 mL) to obtain **5** (12 mg). On the other hand, subfraction EO2.2.4 (45 mg) was subjected to silica gel column chromatography (1.5 g) and eluted with an isocratic elution (CH_2_Cl_2_:CH_3_OH, 95:05) to obtain **5** (17.8 mg). Subfraction EO2.2.5 (25 mg) was subjected to preparative TLC (CH_2_Cl_2_:CH_3_OH, 9:1, 40 mL) to obtain **5** (5 mg) and **6** (4 mg).

#### 4.3.1. Chlorogenic Acid (**1**)

Amorphous powder; ^1^H NMR (700 MHz, D_2_O) δ 7.55 (d, *J* = 15.9 Hz, H-7′), 7.13 (d, *J* = 1.7 Hz, H-2′), 7.04 (dd, *J* = 8.4, 1.4 Hz, H-6′), 6.90 (d, *J* = 8.2 Hz, H-5′), 6.29 (d, *J* = 15.9 Hz, H-8′), 5.29 (td, *J* = 9.2, 4.4 Hz, H-5), 4.27 (q, *J* = 3.5 Hz, H-3), 3.89 (dd, *J* = 8.7, 3.2 Hz, H-4), 2.36–2.17 (m H_2_-2), 2.17–2.03 (m, H_2_-6); ^13^C NMR (175 MHz, D_2_O) δ 177.46 (C-7), 168.66 (C-9′), 147.07 (C-4′), 146.14 (C-7′), 144.21 (C-3′), 126.86(C-1′), 122.65 (C-6′), 116.14 (C-5′), 115.09 (C-2′), 114.36 (C-8′), 75.07 (C-1), 71.53 (C-4), 70.68 (C-5), 69.31 (C-3), 36.51 (C-2, C-6); ESIMS (negative-ion mode) *m*/*z* 352.5 [M − H]^−^.

#### 4.3.2. Rosmarinic Acid (**2**)

Amorphous powder; ${\left[a\right]}_{D}^{20}=$+70.58 (c 0.0017, MeOH) ^1^H NMR (700 MHz, D_2_O) δ 7.23 (d, *J* = 15.9 Hz, H-7), 6.82 (d, *J* = 1.5 Hz, H-2), 6.75 (d, *J* = 8.0 Hz, H-5′), 6.74 (d, *J* = 2.1 Hz, H-2′), 6.68 (d, *J* = 8.2 Hz, H-5), 6.66 (dd, *J* = 8.3, 1.6 Hz, H-6), 6.59 (dd, *J* = 8.2, 1.8 Hz, H-6′), 5.97 (d, *J* = 15.9 Hz, H-8), 5.06 (dd, *J* = 7.2, 5.1 Hz, H-8′), 2.96 (dd, *J* = 14.4, 4.9 Hz, H-7′a), 2.91 (dd, *J* = 14.4, 7.4 Hz, H-7′b); ^13^C NMR (175 MHz, D_2_O) δ 173.9 (C-9′), 168.2 (C-9), 147.0 (C-3), 146.8 (C-7), 143.9 (C-4), 143.7 (C-3′), 142.8 (C-4′), 128.4 (C-1′), 126.5 (C-1), 122.7 (C-6), 121.9 (C-6′), 117.1 (C-2′), 116.0 (C-5′), 115.8 (C-5), 115.1 (C-2), 113.0 (C-8), 73.5 (C-8′), 36.0 (C-7′); ESIMS (negative-ion mode) *m*/*z* 358.5 [M − H]^−^.

#### 4.3.3. Protocatechuic Acid (**3**)

Amorphous powder; ^1^H NMR (700 MHz, Acetone-*d*_6_) δ 7.53 (1H, d, *J* = 2.0 Hz, H-2), 7.47 (1H, dd, *J* = 8.3, 2.0 Hz, H-6), 6.89 (1H, d, *J* = 8.3 Hz, H-5). ^13^C NMR (175 MHz, Acetone) δ 166.55 (C-7), 149.77 (C-4), 144.63 (C-3), 122.71(C-6), 122.23 (C-1), 116.55 (C-2), 114.79 (C-5).

#### 4.3.4. Caffeic Acid (**4**)

Amorphous powder; ^1^H NMR (700 MHz, Acetone-*d*_6_) δ 7.54 (1H, d, *J* = 16.0 Hz, H-7), 7.16 (1H, d, *J* = 2.1 Hz, H-1), 7.04 (1H, dd, *J* = 8.2, 2.1 Hz, H-6), 6.87 (1H, d, *J* = 8.1 Hz, H-5), 6.26 (1H, d, *J* = 15.9 Hz, H-8). ^13^C NMR (175 MHz, Acetone) δ 166.55 (C-9), 147.74 (C-4), 145.39 (C-3), 144.96 (C-7), 126.80 (C-1), 121.54 (C-6), 115.46 (C-5), 114.91 (C-8), 114.26 (C-2).

#### 4.3.5. Kaempferol-3-*O*-(2,6-*di*-*O*-*trans*-*ρ*-coumaryl)-*β*-d-glucopyranoside (**5**)

Amorphous yellow powder;$\;{\left[a\right]}_{D}^{20}=$−137 (c 0.00092, MeOH); UV (MeOH) λmax (log ε) 208 (0.92), 269 (0.44), 314 (0.65) nm; IR (ATR) νmax 3292, 2937, 1651, 1601, 1510, 1161, 1076 cm^−1^; ^1^H NMR (400 MHz, Acetone-*d*6) δ 12.74 (s, OH-5), 8.11 (d, *J* = 8.9 Hz, H-2′, H-6′), 7.73 (d, *J* = 16.0 Hz, H-7‴), 7.59 (d, *J* = 8.6 Hz, H-2‴, H-6‴), 7.46 (d, *J* = 16.0 Hz, H-7⁗), 7.44 (d, *J* = 8.56Hz, H-2⁗, H-6⁗), 6.99 (d, *J* = 8.9 Hz, H-3′, H-5′), 6.91 (d, *J* = 8.61, H-3‴, H-5‴), 6.90 (d, *J* = 8.62, H-3⁗, H-5⁗), 6.45 (d, *J* = 16.0 Hz, H-8‴), 6.44 (d, *J* = 2.1 Hz, H-8), 6.21 (d, *J* = 1.8 Hz, H-6), 6.19 (d, *J* = 16.0 Hz, H-8⁗), 5.92 (d, *J* = 8.1 Hz, H-1″), 5.16 (dd, *J* = 9.5, 8.1 Hz, H-2″), 4.42 (dd, *J* = 11.9, 2.1 Hz, H-6a″), 4.22 (dd, *J* = 11.9, 6.2 Hz, H-6b″), 3.84 (q, *J* = 9.0 Hz, H-3″), 3.75–3.64 (m, H-5″), 3.56(dd, *J* = 9.68, 8.94 Hz, H-4″). ^13^C NMR (100 MHz, Acetone) δ 177.79 (C4), 166.3 (C9⁗), 165.93 (C9‴), 164.0 (C7), 162.2 (C5), 159.9 (C4′), 159.7 (C4‴), 159.6 (C4⁗), 157.0 (C2), 156.8 (C9), 145.1 (C7‴), 144.6 (C7⁗), 133.2 (C3), 131.1 (C2′,C6′), 130.1 (C2‴, C6‴, C2⁗, C6⁗), 126.2 (C1‴), 126.0 (C1⁗), 121.9 (C1′), 115.82 (C3‴, C5‴), 115.8 (C3⁗, C5⁗), 115.10 (C3′, C5′), 114.8 (C8‴), 114.23 (C8⁗), 104.7 (C10), 98.9 (C1″), 98.7 (C6), 93.60 (C8), 75.0 (C3″), 74.6 (C5″), 74.11 (C2″), 70.81 (C4″), 62.79 (C6″); ESIMS (positive-ion mode) *m*/*z* 740.7 [M]^+^.

#### 4.3.6. Quercetin-3-*O*-(2,6-*di*-*O*-*trans*-*ρ*-coumaryl)-*β*-d-glucopyranoside (**6**)

Amorphous yellow powder; ${\left[a\right]}_{D}^{20}=$ −140 (c 0.0025, MeOH); UV (MeOH) λmax (log ε) 231 (2.5), 313 (1.22) nm; IR (ATR) νmax 3254, 2924, 2854, 1625, 1571, 1485, 1443, 1320, 1198, 1154 cm^−1^; ^1^H NMR (700 MHz, Methanol-*d*_4_) δ 7.73 (d, *J* = 15.9 Hz, H-7‴), 7.56 (d, *J* = 2.1 Hz, H-2′), 7.55 (dd, *J* = 8.6, 2.1 Hz, H-6′), 7.50 (d, *J* = 8.7 Hz, H-2‴, H-6‴), 7.42 (d, *J* = 15.9 Hz, H-7⁗), 7.33 (d, *J* = 8.6 Hz, H-2⁗, H-6⁗), 6.85 (d, *J* = 9.0 Hz, H-5′), 6.83 (d, *J* = 8.6 Hz, H-3⁗, H-5⁗), 6.44 (d, *J* = 15.9 Hz, H-8″), 6.27 (d, *J* = 2.1 Hz, H-8), 6.10 (d, *J* = 15.9 Hz, H-8⁗), 6.09 (d, *J* = 2.0 Hz, H-6), 5.63 (d, *J* = 8.0 Hz, H-1″), 5.13 (dd, *J* = 9.5, 8.0 Hz, H-2″), 4.38 (dd, *J* = 11.7, 2.2 Hz, H-6a″), 4.26 (dd, *J* = 11.8, 6.8 Hz, H-6b″), 3.68 (t, *J* = 9.2 Hz, H-3″), 3.56 (ddd, *J* = 9.3, 6.9, 2.3 Hz, H-5″), 3.46 (m, H-4);^13^C NMR (175 MHz, Methanol-*d4*) δ 179.2 (C4), 168.8 (C9⁗), 168.6 (C9‴), 165.7 (C7), 163.0 (C5), 161.3 (C4‴), 161.2 (C4⁗), 158.8 (C2), 158.3 (C9), 149.7 (C4′), 147.1 (C7‴), 146.6 (C7⁗), 146.0 (C3′), 134.6 (C3), 131.3 (C2⁗, C6⁗), 131.2 (C2‴, C6‴), 127.3 (C1⁗), 127.1 (C1‴), 123.5 (C6′), 123.1 (C1′), 117.1 (C2′), 116.8 (C3⁗, C5⁗), 116.8 (C3‴, C5‴), 116.0 (C5′), 115.2 (C8‴), 114.7 (C8⁗), 105.7 (C10), 100.4 (C1″), 99.8 (C6), 94.6 (C8), 76.2 (C3″), 76.0 (C5″), 75.7 (C2″), 72.0 (C4″), 64.2 (C6″); ESIMS (negative-ion mode) *m*/*z* 738.6 [M − H_2_O + H]^−^.

### 4.4. HPLC Analysis

The HPLC profiles were developed using an Agilent 1260 HPLC instrument with the specification mentioned in Section 2.1. The separation was carried out using a Phenomenex^®^ Kinetex C_18_ 100 Å (50 × 2.1 mm id., 5 μm) reversed-phase column. The column temperature was kept at 35 °C. Working solutions of samples were prepared by dissolving 10, 1 and 1 mg of extracts of *E-cymosum* (WE and BuS), isolated compounds, and standards, respectively, with the appropriate solvent for each (1mL of H_2_O for WE and 1 mL of MeOH for BuS, compounds and standards). All samples were filtered on membrane filters (PTFE, 0.20 μm) and injected (2 μL). Elution was carried out with water containing 0.1% (*v*/*v*) formic acid as solvent A and acetonitrile (MeCN) as solvent B, starting with a mixture of 99% of A and 1% of B, increasing the amount of solvent B as follows: 25% at 14 min, 30% at 14–18 min, 35% at 18–22 min, 95% at 22–27, holding this mixture for a minute and returning to the initial conditions at 30 min. The flow rate was 0.35 mL/min. For UV detection, the wavelength program was set at an acquisition of λ 240, 254, 280, 320 and 365 nm; the UV spectra were recorded from 230 at 400.

### 4.5. In Vitro Assays

#### 4.5.1. Inhibition Assay of Glucose-6-phosphatase (G6Pase)

Rat liver microsomal fractions isolated through differential centrifugation were diluted 1:1 in buffer (40 mM imidazole, 250 mM sucrose, pH 7) and added to reaction mixtures, containing buffer and different concentrations of inhibitor samples (chlorogenic acid or compounds). Enzymatic activity started by adding 20 mM of glucose-6-phosphate (G6P). Next, the mixtures were incubated at 22 °C for 20 min and ended with the addition of 900 μL of a solution of 0.42% ammonium molybdate in 1 N H_2_SO_4_, 10% SDS, and 10% ascorbic acid. Finally, they were incubated at 45 °C for 20 min and measured at 830 nm.

#### 4.5.2. Inhibition Assay of Fructose-1,6-bisphosphatase (FBPase)

Buffer (5 μM EDTA, 5 mM MgCl_2_, 50 mM Tris-HCl, pH 7.2) enriched with 0.1 mM of fructose-1,6-bisphosphate was added and incubated for 5 min with different concentrations of inhibitor or samples (adenosine 5′-monophosphate or compounds) in reaction mixtures of 1 mL. Enzymatic activity started by adding rat liver cytosolic supernatant diluted 9:1 and, 15 min later, ended with the addition of a color reagent previously prepared by mixing 1 volume of 7.5% ammonium molybdate and 0.17% TWEEN 20 with 4 volumes of stock solution (0.12% malachite green in 5 volumes of water with 1 volume of H_2_SO_4_). Finally, the reaction mixtures were incubated for 10 min at room temperature and measured at 630 nm.

## 5. Conclusions

This work is the first report on isolation of chlorogenic acid (**1**), rosmarinic acid (**2**), caffeic acid (**3**), protocatechuic acid (**4**), kaempferol-3-*O*-(2,6-*di*-*O*-*trans*-*ρ*-coumaryl)-*β*-d-glucopyranoside (**5**), and quercetin-3-*O*-(2,6-*di*-*O*-*trans*-*ρ*-coumaryl)-*β*-d-glucopyranoside (**6**) from the aerial parts of *Eryngium cymosum*. Moreover, this is the first report of the isolation and elucidation of quercetin-3-*O*-(2,6-*di*-*O*-*trans*-*ρ*-coumaryl)-*β*-d-glucopyranoside (**6**). Recently, the acute hypoglycemic effect of the aqueous extract of *Eryngium cymosum* has been proved [6]. Our results support the previous observation, the inhibitory effect of compound **5** over G6Pase and FBPase could play an important role in the hypoglycemic effect of the plant in the fasting state, but it is possible that the six isolated compounds play a major role in the overall hypoglycemic effect of the extract since they could act in a synergic way. More experiments are required to corroborate it.

## Figures and Tables

**Table 1:** Spectral data of compound **6**.

	6	
Position	δ_H_, Multiplicity, (*J* in Hz)	δ_C_
2	-	158.8
3	-	134.6
4	-	179.2
5	-	163.0
6	6.09, d, *J* = 2.0 Hz	99.8
7		165.7
8	6.27, d, *J* = 2.1 Hz	94.6
9	-	158.3
10	-	105.7
1′	-	123.1
2′	7.56, d, *J* = 2.0 Hz	117.1
3′	-	146.0
4′	-	149.7
5′	6.85, d, *J* = 9.0 Hz	116.0
6′	7.55, dd, *J* = 2.1, 8.6 Hz	123.5
1″	5.63, d, *J* = 8.0 Hz	100.4
2″	5.13, dd, *J* = 9.5, 8.0 Hz	75.7
3″	3.68, t, *J* = 9.2 Hz	76.2
4″	3.46, m	72.0
5″	3.56, ddd, *J* = 9.3, 6.9, 2.3 Hz	76.0
6″	4.38, dd, *J* = 11.7, 2.2 Hz, H-6a 4.22, dd, *J* = 11.8, 6.8 Hz, H6b	64.2
1‴	-	127.1
2‴, 6‴	7.50, d, *J* = 8.7 Hz	131.2
3‴, 5‴	6.83, d, *J* = 8.6 Hz	116.8
4‴	-	161.3
7‴	7.73, d, *J* = 15.9 Hz	147.1
8‴	6.44, d, *J* = 15.9 Hz	115.2
9‴	-	168.6
1⁗	-	127.3
2⁗, 6⁗	7.33, d, *J* = 8.6 Hz	131.3
3⁗, 5⁗	6.83, d, *J* = 8.6, Hz	116.8
4⁗		161.2
7⁗	7.42, d, *J* = 15.9 Hz	146.6
8⁗	6.10, d, *J* = 15.9 Hz	114.7
9⁗		168.8

**Table 2:** Inhibitory activity of identified compounds on enzymes involved in glucose-producing pathways.

Compound	G6Pase IC_50_ (μg/mL)	FBPase IC_50_ (μg/mL)
Caffeic acid (4)	719.8 ± 192.9	-
kaempferol-3-O-(2,6-di-O-trans-ρ-coumaryl)-β-d-glucopyranoside (5)	27.7 ± 1.9	52.5 ± 6.4
Chlorogenic acid *	179.7 ± 27.5	-
Adenosine 5′-monophosphate ^+^	-	16.9 ± 2.2

IC_50_ values expressed as mean *±* SEM of two assays in duplicate. G6Pase: glucose-6-phosphatase. FBPase: fructose-1,6-bisphosphatase. * G6Pase positive control. ^+^ FBPase positive control.

## Data Availability

The data is contained within the article and supplementary file.

## Supplementary Materials

The following supporting information can be downloaded at: https://www.mdpi.com/article/10.3390/plants11070992/s1, Figure S1: 1H NMR spectrum of compound **5** in 400 MHz at CD3COCD3; Figure S2: 13C NMR spectrum of compound **5** in 100 MHz at CD3COCD3; Figure S3: ESI-MS of compound **5**; Figure S4: 1H NMR spectrum of compound **6** in 700 MHz at CD3OD; Figure S5: COSY spectrum of compound **6** in 700 MHz at CD3OD; Figure S6: HSQC spectrum of compound **6** in CD3OD; Figure S7: HMBC spectrum of compound **6** in CD3OD; Figure S8: 13C NMR spectrum of compound **6** in 175 MHz at CD3OD; Figure S9: ESI-MS of compound **6**; Figure S10: IR spectrum of compound **6**.
