# Supplementary material for: Phytochemical Study of Eryngium cymosum F. Delaroche and the Inhibitory Capacity of Its Main Compounds on Two Glucose-Producing Pathway Enzymes
Source: Plants (Basel). 2022 Apr 5;11(7):992. doi: 10.3390/plants11070992 (PMC9002393; doi:10.3390/plants11070992)
Supplement: Supplementary file 1 [file plants-11-00992-s001.zip › plants-1621393-supplementary.pdf]

Phytochemical study of Eryngium cymosum F. Delaroche and the inhibitory capacity of its main compounds on two glucose-producing pathway enzymes.

Adriana Romo-Pérez, Sonia Marlen Escandón-Rivera, Luis D. Miranda and Adolfo Andrade-Cetto.

Supporting Information

Table of Contents

**Figure S1**. ^{1}H NMR spectrum of compound 5 in 400 MHz at CD_3OD.	3
**Figure S2**. ^{13}C NMR spectrum of compound 5 in 100 MHz at CD_3OD.	4
**Figure S3.** ESI-MS of compound 5.	5
**Figure S4.** ^{1}H NMR spectrum of compound 6 in 700 MHz at CD_3OD.	6
**Figure S5.** COSY spectrum of compound 6 in 700 MHz at CD_3OD.	7
**Figure S6**. HSQC spectrum of compound 6 in CD_3OD.	8
**Figure S7.** HMBC spectrum of compound 6 in CD_3OD.	9
**Figure S8.** ^{13}C NMR spectrum of compound 6 in 175 MHz at CD_3OD.	10
**Figure S9.** ESI-MS of compound 6.	11
**Figure S10.** IR spectrum of compound 6.	12

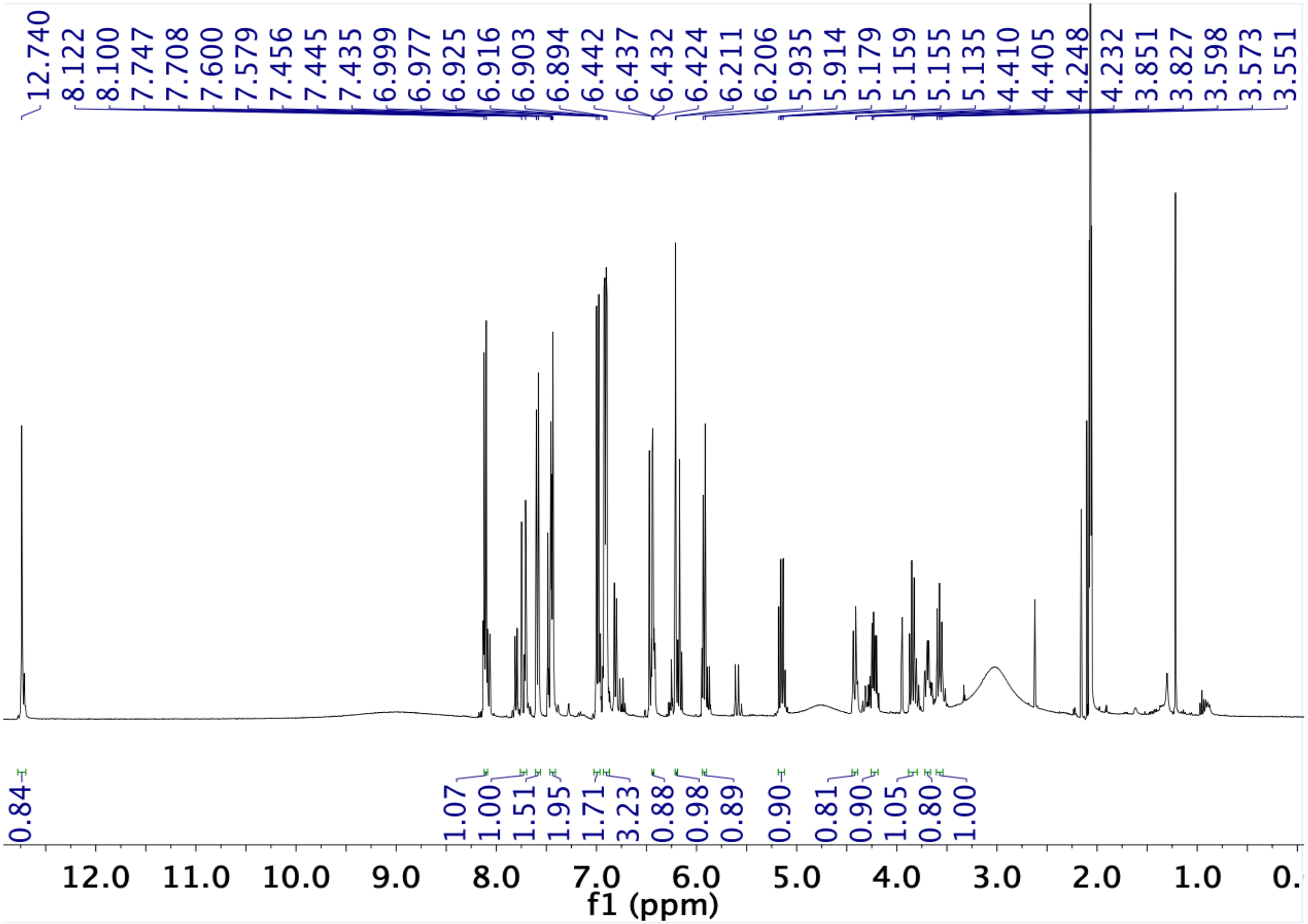

Figure S1. ^{1}H NMR spectrum of compound 5 in 400 MHz at CD_3COCD_3.

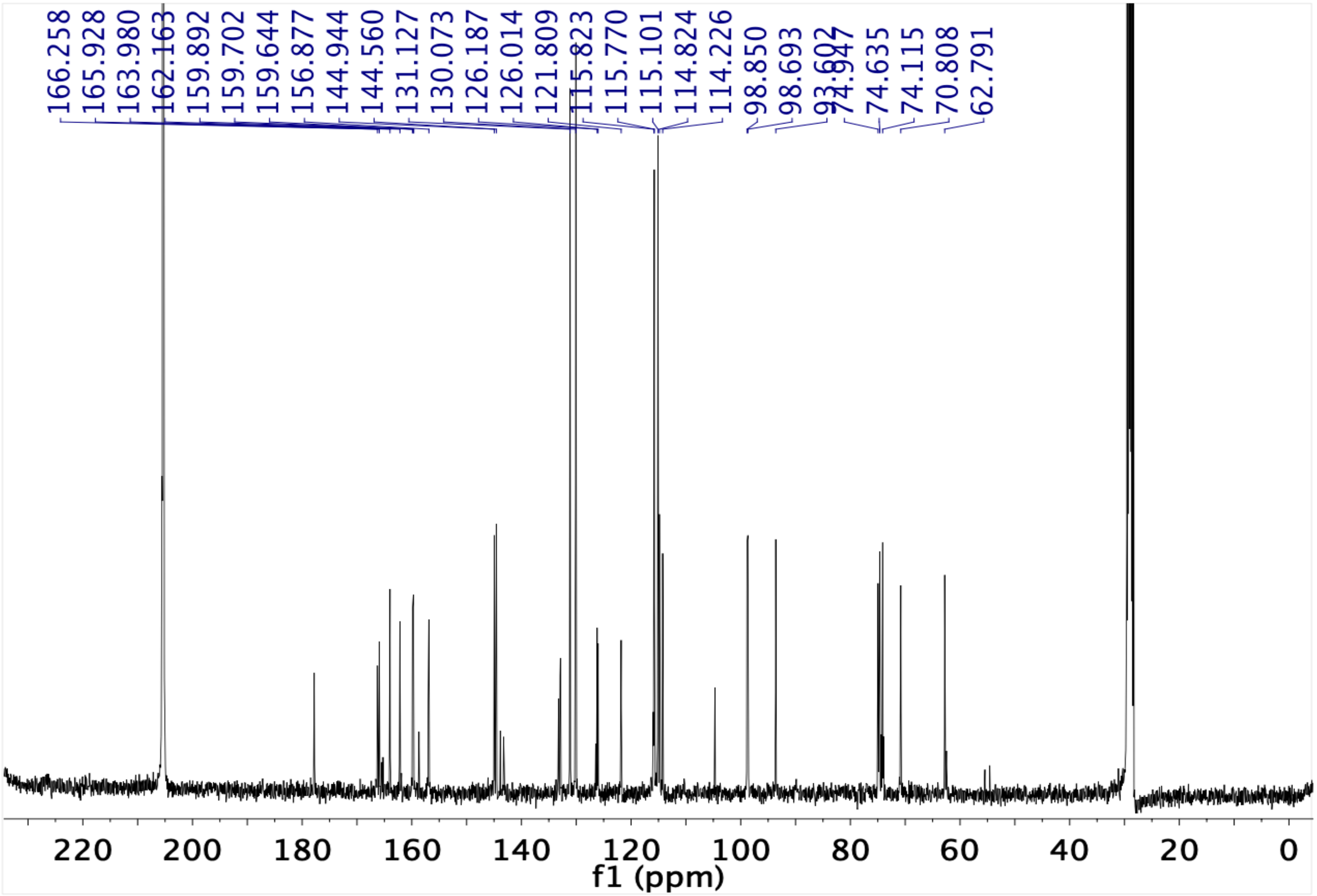

Figure S2. ^{13}C NMR spectrum of compound 5 in 100 MHz at CD_3COCD_3

Compound Mass Spectrum List Report

Analysis Info

Analysis Name	D:\Data\mgl\mgl00060.d	Acquisition Date	19/08/2021 10:27:08 a.m.
Method	Secretaria Técnica.m	Operator	Carmen Marquez
Sample Name	ARPEcMPBK	Instrument	esquire6000
Comment	210221-mgl-03<br>HPLC-MS		

Acquisition Parameter

Ion Source Type	ESI	Ion Polarity	Positive	Alternating Ion Polarity	off
Mass Range Mode	Std/Normal	Scan Begin	100 m/z	Scan End	1500 m/z
Capillary Exit	142.4 Volt	Skim 1	40.0 Volt	Trap Drive	83.8
Accumulation Time	21710 µs	Averages	10 Spectra	Auto MS/MS	off

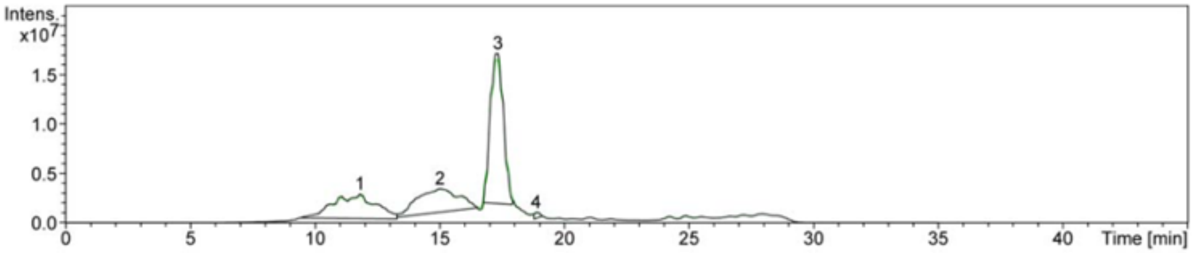

#	RT [min]	Area
1	11.8	293249841
2	15.0	277616116
3	17.3	558703119
4	18.9	8442937

Cmpd 3, 17.3 min

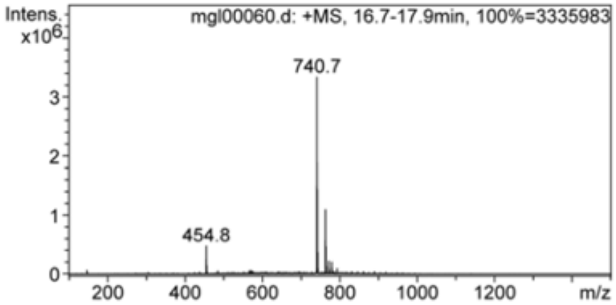

#	m/z	I
1	454.8	476873
2	455.8	136903
3	740.7	3335983
4	741.7	1431699
5	742.7	368610
6	762.7	1097236
7	763.7	462938
8	764.7	131573
9	770.7	221876
10	778.7	206928

Figure S3. ESI-MS of compound 5.

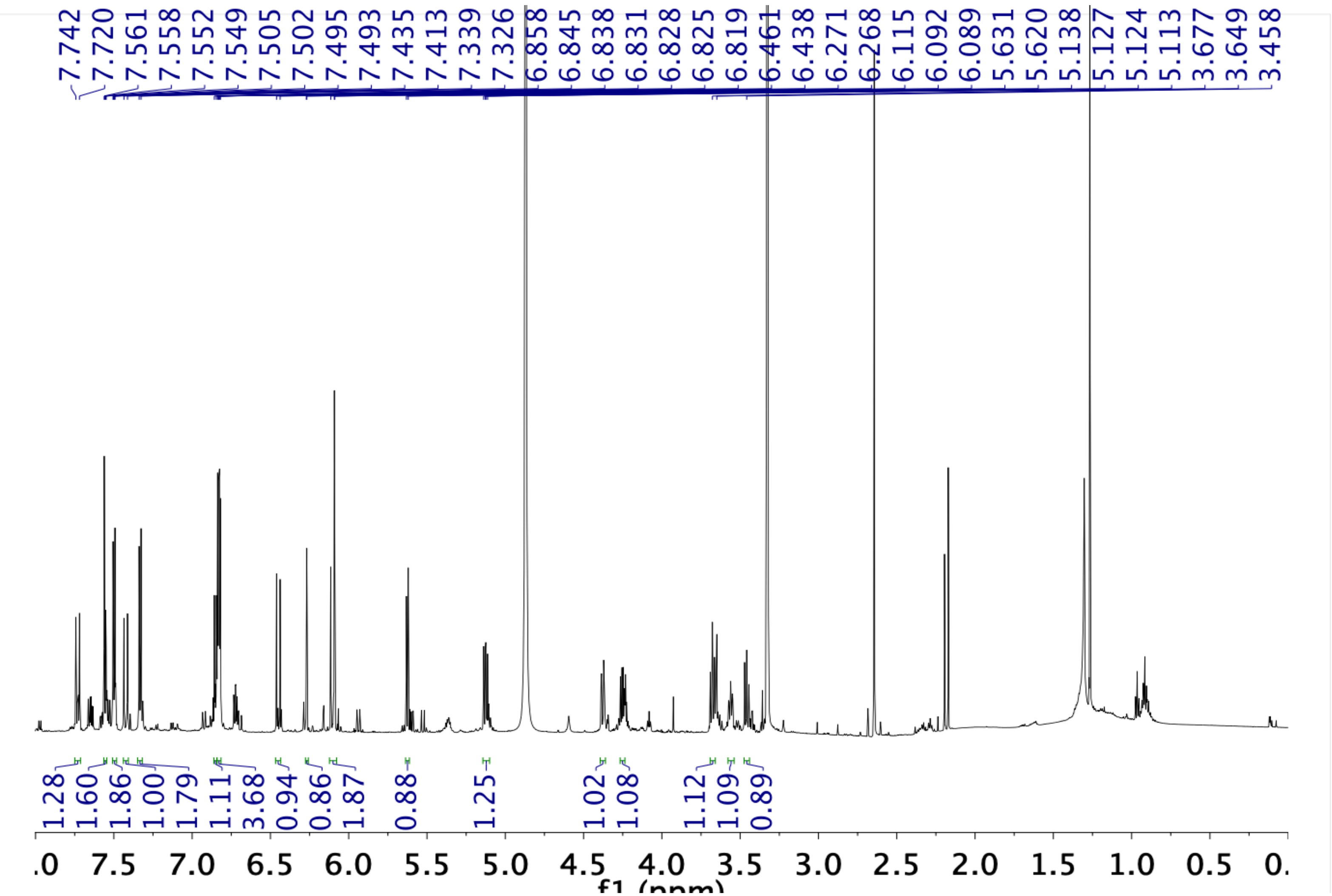

Figure S4. ^{1}H NMR spectrum of compound 6 in 700 MHz at CD_3OD.

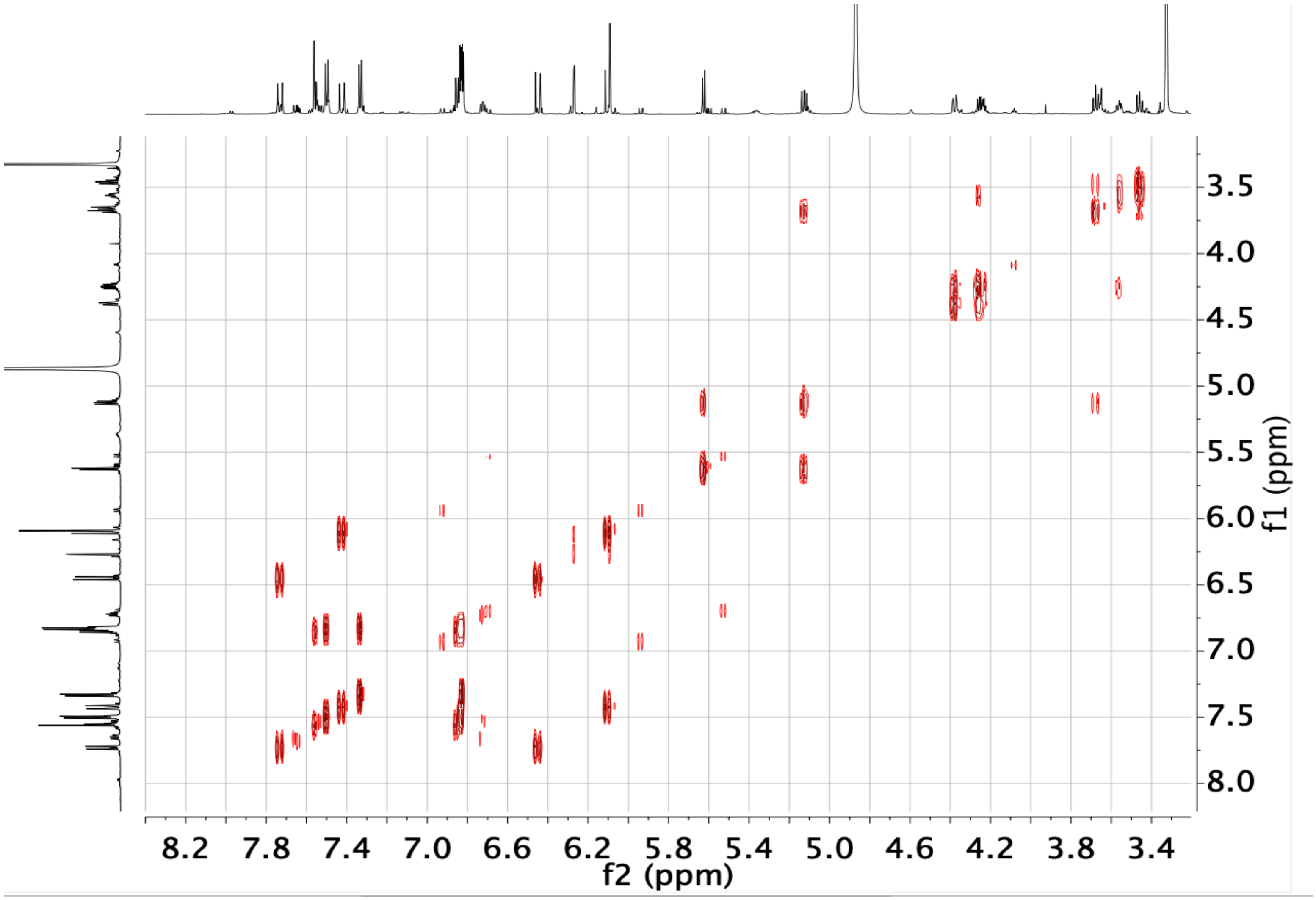

Figure S5. COSY spectrum of compound 6 in 700 MHz at CD_3OD

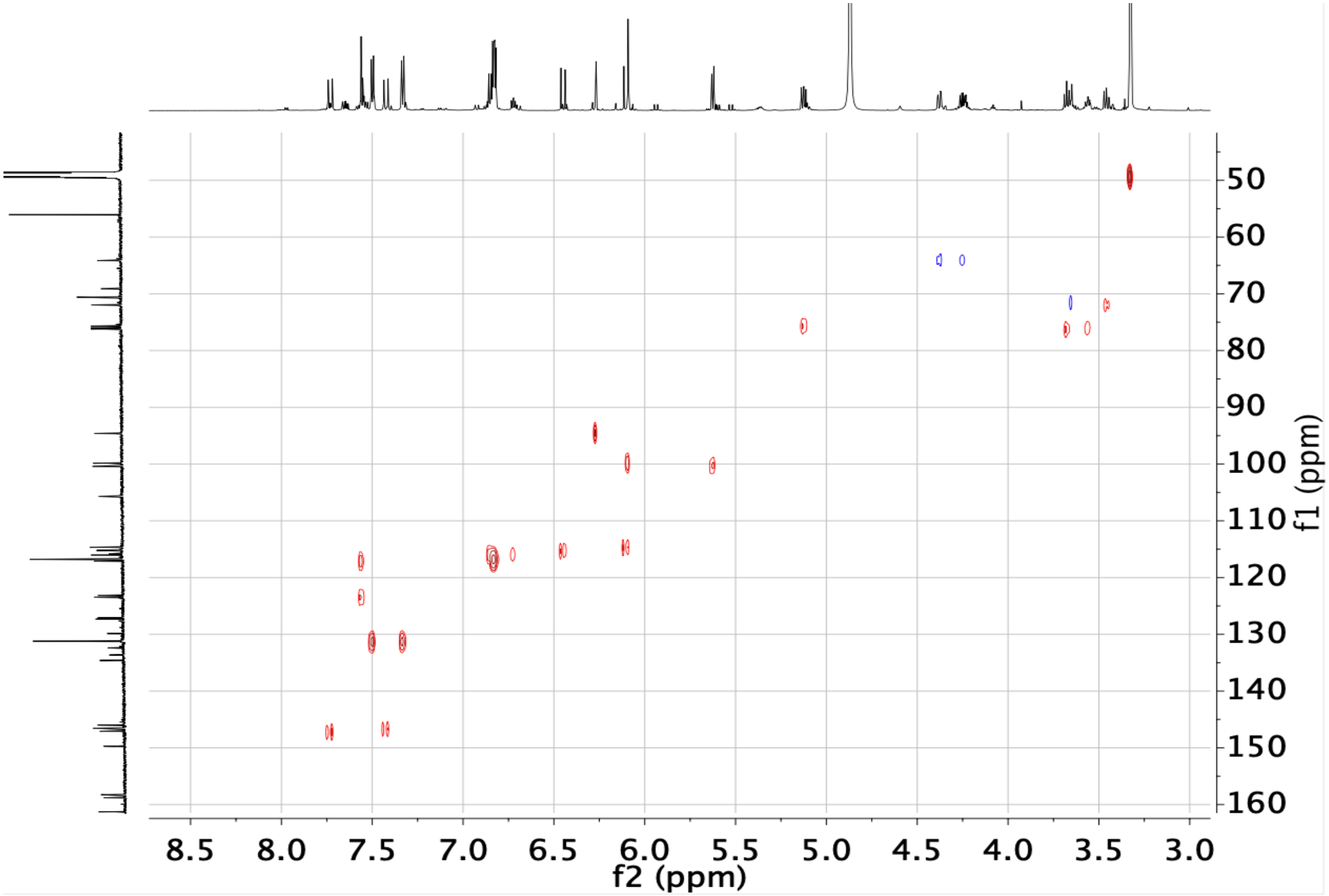

Figure S6. HSQC spectrum of compound 6 in in CD_3OD.

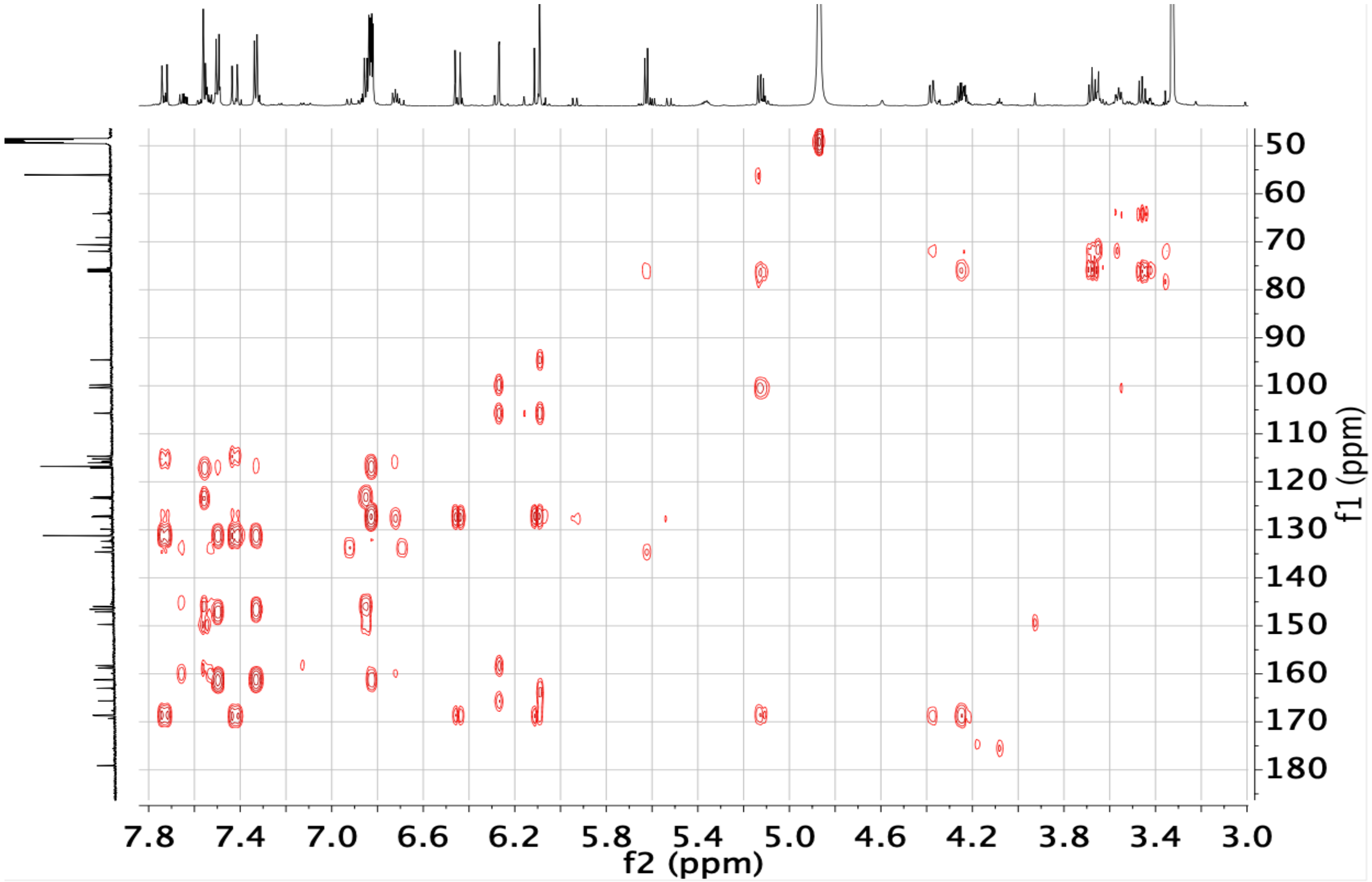

Figure S7. HMBC spectrum of compound 6 in CD_3OD.

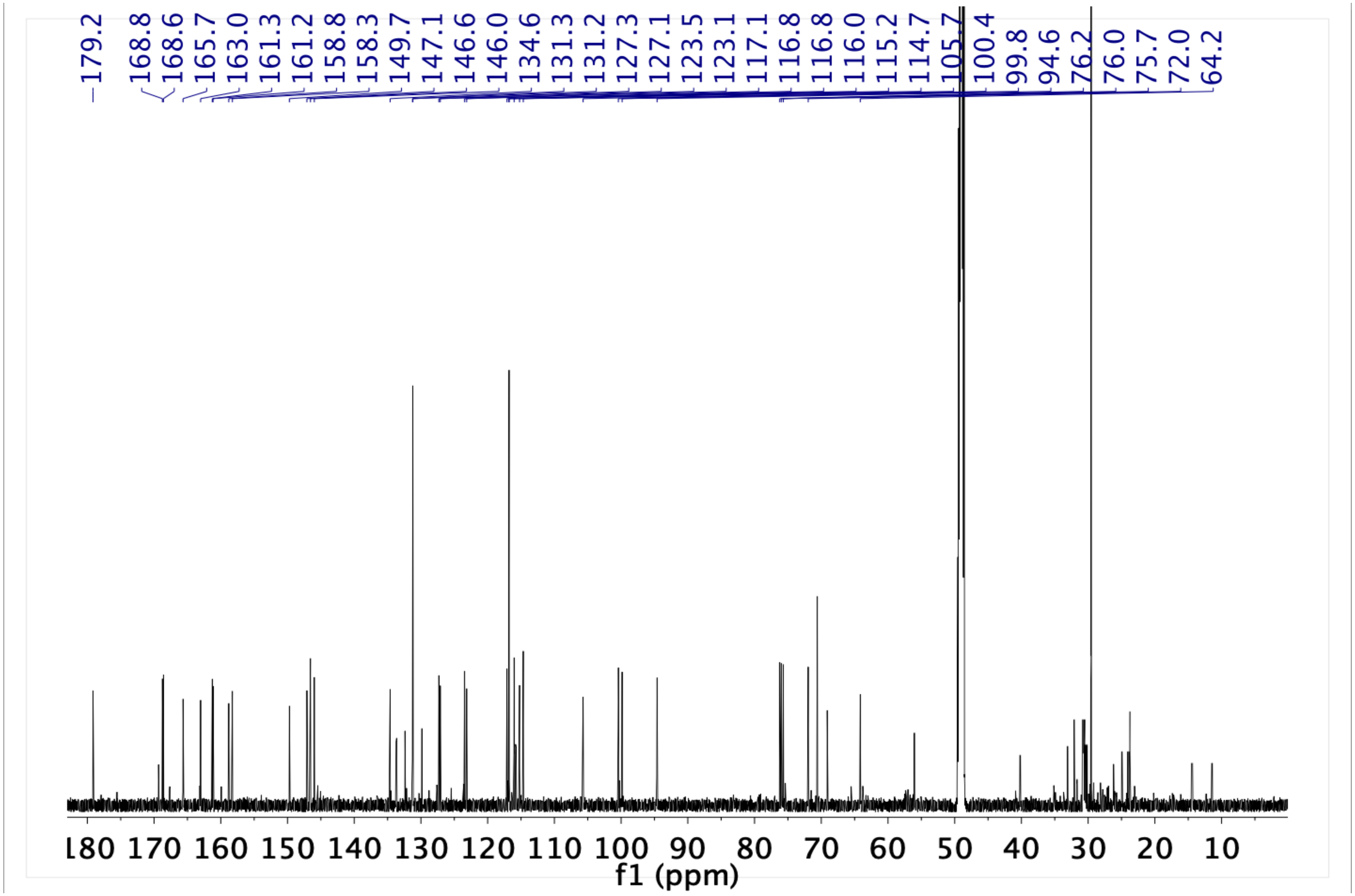

Figure S8. ^{13}C NMR spectrum of compound 6 in 175 MHz at CD_3OD.

Compound Mass Spectrum List Report

Analysis Info

Analysis Name	D:\Data\mgl\mgl00061.d	Acquisition Date	19/08/2021 11:16:13 a.m.
Method	Secretaria Técnica.m	Operator	Carmen Marquez
Sample Name	ARPEcl 2-2-2-5-2pp	Instrument	esquire6000
Comment	210221-mgl-04<br>HPLC-MS		

Acquisition Parameter

Ion Source Type	ESI	Ion Polarity	Positive	Alternating Ion Polarity	off
Mass Range Mode	Std/Normal	Scan Begin	100 m/z	Scan End	1500 m/z
Capillary Exit	142.4 Volt	Skim 1	40.0 Volt	Trap Drive	83.8
Accumulation Time	21328 µs	Averages	10 Spectra	Auto MS/MS	off

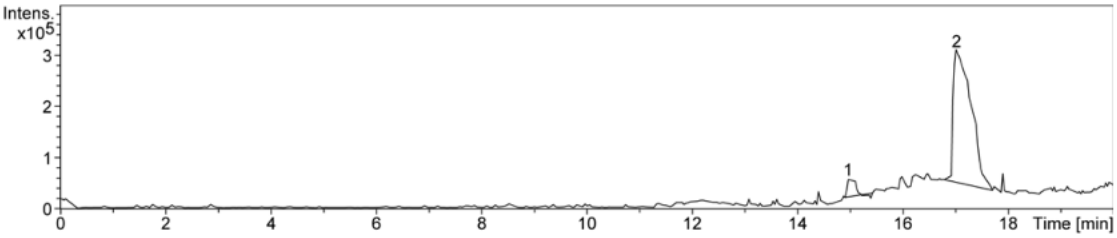

#	RT [min]	Area
1	15.0	461648
2	17.0	6145050

Cmpd 2, 17.0 min

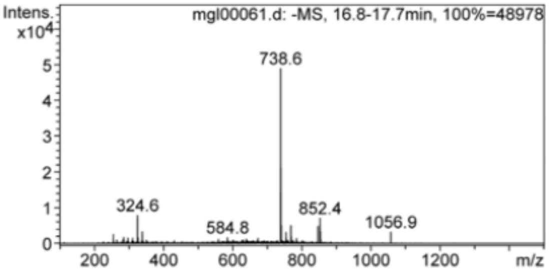

#	m/z	I
1	324.6	7953
2	338.6	3353
3	738.6	48978
4	739.5	21062
5	740.5	5151
6	768.5	5097
7	846.4	4902
8	852.4	7100
9	853.5	3343
10	1056.9	3260

Figure S9. ESI-MS of compound 6.

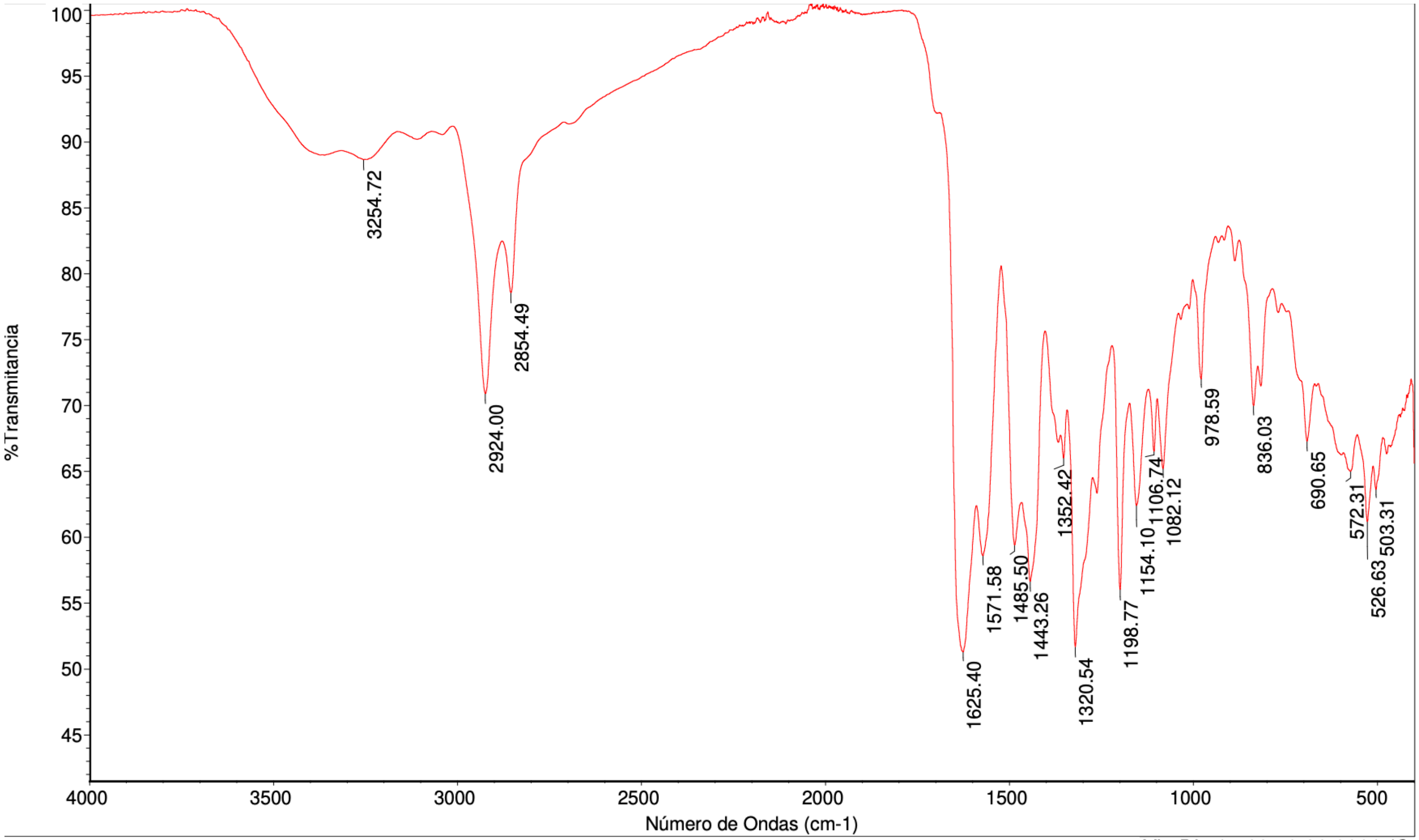

Figure S10. IR Spectrum of compound 6.
